# Differential neutrophil responses to bacterial stimuli: Streptococcal strains are potent inducers of heparin-binding protein and resistin-release

**DOI:** 10.1038/srep21288

**Published:** 2016-02-18

**Authors:** Johanna Snäll, Anna Linnér, Julia Uhlmann, Nikolai Siemens, Heike Ibold, Marton Janos, Adam Linder, Bernd Kreikemeyer, Heiko Herwald, Linda Johansson, Anna Norrby-Teglund

**Affiliations:** 1 Department of Medicine Huddinge, Centre for Infectious Medicine, Karolinska Institutet, Karolinska University Hospital, S-141 86 Stockholm, Sweden; 2 Department of Clinical Sciences, Lund University, S-221 84 Lund, Sweden; 3 Institute of Medical Microbiology, Virology, Hygiene and Bacteriology, Rostock University Medical Centre, 18055 Rostock, Germany

## Abstract

Neutrophils are critical for the control of bacterial infections, but they may also contribute to disease pathology. Here we explore neutrophil responses, in particular the release of sepsis-associated factors heparin-binding protein (HBP) and resistin in relation to specific bacterial stimuli and sepsis of varying aetiology. Analyses of HBP and resistin in plasma of septic patients revealed elevated levels as compared to non-infected critically ill patients. HBP and resistin correlated significantly in septic patients, with the strongest association seen in group A streptococcal (GAS) cases. *In vitro* stimulation of human neutrophils revealed that fixed streptococcal strains induced significantly higher release of HBP and resistin, as compared to *Staphylococcus aureus* or *Escherichia coli*. Similarly, neutrophils stimulated with the streptococcal M1-protein showed a significant increase in co-localization of HBP and resistin positive granules as well as exocytosis of these factors, as compared to LPS. Using a GAS strain deficient in M1-protein expression had negligible effect on neutrophil activation, while a strain deficient in the stand-alone regulator MsmR was significantly less stimulatory as compared to its wild type strain. Taken together, the findings suggest that the streptococcal activation of neutrophils is multifactorial and involves, but is not limited to, proteins encoded by the FCT-locus.

While in bacterial infections, the recruitment and activation of neutrophils are critical for the immune defence; it is becoming increasingly evident that neutrophils may also contribute to immunopathology. On one hand, neutrophils play an important role in bacterial killing through mechanisms such as phagocytosis, formation of extracellular traps and production of antimicrobial effector molecules. On the other hand, the release of granule proteins can cause cell and tissue damage to the host, as well as dysregulated inflammatory response^1^.

In sepsis, several studies have shown that neutrophil responses are aberrant with respect to survival, migratory capacity and functionality^2^,^3^,^4^. Whereas many previous studies have focused on the detrimental role of dysregulated migration and impaired phagocytic killing, less is known about the contribution of neutrophils to the hyperinflammatory responses that characterize sepsis and its complications. A distinct feature of sepsis is the recruitment of immature neutrophils from the bone marrow into the circulation. A recent report found circulating neutrophils in sepsis patients to have a suppressed apoptosis, a longer life span and pro-inflammatory phenotype with increased TNFα/IL10 ratio^5^.

Heparin-binding protein (HBP) and resistin are two neutrophil-derived effector molecules that both have been reported to be associated with severity of sepsis^6^,^7^. HBP (also referred to as azurocidin or CAP37) is an inactive serine protease stored within azurophilic granules and secretory vesicles^8^. It has been associated with many functions including antimicrobial and immostimulatory activity (reviewed in^9^). Importantly, as a potent inducer of vascular leakage, HBP has been suggested to be a central player in the pathophysiology of circulatory failure and hypotension^10^,^11^, thereby complicating severe infections. Resistin, a cysteine-rich adipocytokine, has in humans been identified as a potent pro-inflammatory molecule associated with acute and chronic inflammatory conditions^12^,^13^,^14^. We reported on resistin as a marker of severity of sepsis, with a sustained secretion profile compared to early cytokines and the highest systemic resistin levels noted in patients with septic shock^6^. Neutrophils were identified as a novel dominant source of resistin in sepsis patients^15^.

Group A streptococcus (GAS) is one pathogen that has been demonstrated to have a significant impact on neutrophils, by inducing an altered apoptotic differentiation program^16^ and triggering degranulation^17^,^18^. Considering the severity of fulminant invasive GAS infections, such as streptococcal toxic shock syndrome (STSS) and necrotizing fasciitis, it seems likely that neutrophils are key contributors to the multifaceted disease pathophysiology involving hyper-inflammation and pronounced vascular leakage; to perhaps a greater extent than in the case of invasive infections caused by other bacteria.

In this study we explore whether neutrophil responses, in particular the release of the sepsis-associated factors HBP and resistin, differ depending on stimuli and how this relates to sepsis of varying aetiology. To address this, neutrophil stimulation and activation was studied *in vitro* using primary human neutrophils exposed to clinical sepsis isolates or bacterial antigens, as well as through analyses of patients with severe acute bacterial infections.

## Results

### HBP and resistin responses in sepsis patients of varying aetiology

**Figure 1:**
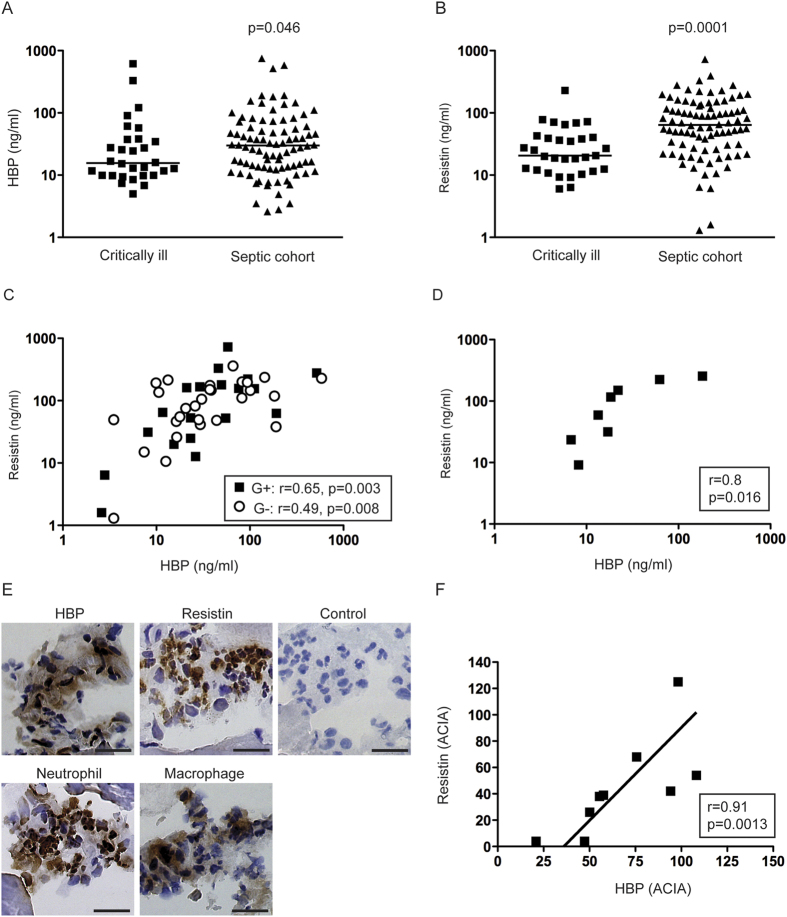
Systemic and local HBP and resistin responses in septic patients. HBP and resistin levels in plasma collected from patients on the day of inclusion were measured with ELISA, for details see Experimental procedures. (**A**,**B**) HBP and resistin levels in plasma from patients with severe sepsis/septic shock (n = 88) or non-infected critically ill patients (n = 31). (**C**) Plasma HBP and resistin levels in severe sepsis/septic shock patients with confirmed Gram-positive (G+) or Gram-negative (G−) infections (n = 48). (**D**) Systemic HBP and resistin in STSS patients (n = 8). The correlation was determined by Pearson’s correlation test, indicated by *p* and *r*-values. (**E**) HBP, resistin, macrophages and neutrophils were analysed by immunohistochemical staining of cryosections of snap-frozen tissue biopsies (n = 9) from patients with severe soft tissue infections caused by GAS. A control staining where the primary antibody was omitted was also performed (control). A representative tissue biopsy is shown. The scale bar indicates 50 μm. (**F**) The stainings were evaluated by microscope and acquired computerized image analysis (ACIA), for details see Experimental procedures. Significant correlation was determined by Spearman test, indicated by *p*- and r-values.

First we measured HBP and resistin levels in acute phase plasma of patients with severe sepsis/septic shock caused by different bacteria (Fig. 1). The results showed that septic patients, including both Gram-positive and Gram-negative bacterial infections, had significantly higher levels of both factors when compared to non-infected critically ill patients (Fig. 1A,B). There were no significant differences in HBP or resistin levels between patients infected with Gram-positive (n = 20) or Gram-negative (n = 28) bacteria (Fig. 1C). However, the Gram-positive cohort demonstrated a stronger correlation between HBP and resistin (r = 0.65, p = 0.003) as compared to Gram-negative cohort (r = 0.49, p < 0.001). The Gram-positive infections were predominantly caused by *Enterococcus* or *Staphylococcus aureus,* but did not include any streptococcal infections^19^. As GAS has been reported to have a strong impact on neutrophils^15^,^16^,^18^, a separate patient cohort consisting of streptococcal septic shock patients, i.e. GAS STSS patients, was also analysed. Similarly to the septic shock cohort, high levels of both HBP and resistin were detected in plasma of STSS patients, and demonstrated an even stronger correlation (r = 0.8, p = 0.016) (Fig 1D).

To further validate the finding that GAS infected patients had such strong correlation between systemic HBP and resistin, studies were undertaken to analyse these responses at the local site of infection, i.e. in infected soft tissue. Data were available from previously analysed snap-frozen tissue biopsies collected from patients with GAS necrotizing fasciitis or severe cellulitis^15^,^20^. The tissue biopsies had been analysed for expression of either HBP or resistin as well as phagocyte infiltration by intracellular immunohistochemical stainings. As previously reported, macrophages, neutrophils, HBP and resistin were detected in all infected biopsies (Fig. 1E). This can be compared to only a few resistin positive cells found in skin biopsies from healthy controls^15^,^20^. In agreement with the above-presented data from circulation, a correlation test revealed a significant correlation between HBP and resistin at the infected tissue site (r = 0.91, p = 0.0013) (Fig. 1F).

### Additive effect of HBP and resistin on inflammatory response

**Figure 2:**
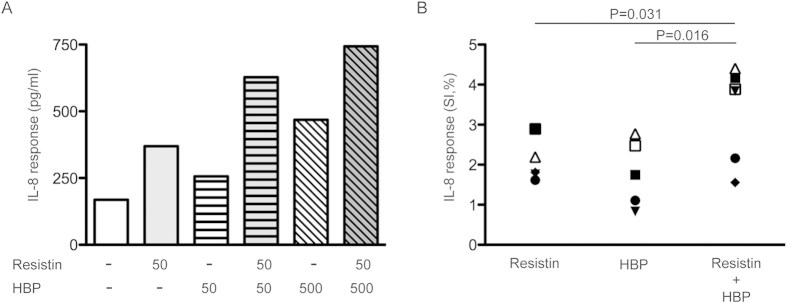
HBP and resistin induce a significant inflammatory response *in vitro*. PBMCs of healthy donors were stimulated with different combinations of HBP (50 or 500 ng/ml) and resistin (50 ng/ml) alone, or the combination of the two proteins. Luminex analysis of IL-8 levels of the cell culture supernatants was performed. (**A**) Result of one experiment using cells from one donor. (**B**) Result of six donors stimulated with HBP (500 ng/ml) and resistin (50 ng/ml), alone or combined, are shown; each donor indicated with a different symbol. IL-8 responses are here shown in stimulation index (SI, %), were the stimulated value is divided with the unstimulated value. Mann-Whitney *U* test was used for comparison between groups and differences are shown in *p* values.

Next, we tested both antimicrobial and immunostimulatory properties of HBP and resistin. Antibacterial activity against GAS, *S. aureus* and *E. coli* was assessed using a Bioscreen C Microbiological Growth analyser measuring the turbidity in cultures containing a concentration range of 0.1–5 μg/ml HBP or 0.1–2 μg/ml resistin. Similar OD curves were observed for GAS and *S. aureus* in the presence or absence of resistin or HBP; thus, excluding any antimicrobial effect. In accordance with the previously reported antimicrobial activity of HBP against Gram-negative bacteria^9^, *E coli* showed a slight reduction in OD during log-phase (mean % reduction: 14 ± 2), while no change in growth when resistin was added. As both HBP and resistin have been reported to exert pro-inflammatory activities^9^,^10^,^12^,^13^,^14^, it was of interest to study whether there are potential additive or synergistic effects of these factors. For this purpose, PBMCs from healthy donors were stimulated with either HBP or resistin, or the combination of the two proteins. IL-8, a classical sepsis associated pro-inflammatory cytokine, was measured in culture supernatants. As shown in Fig. 2, IL-8 levels were significantly higher in supernatants from cells stimulated with the combination of HBP and resistin, as compared to each protein alone (p ≥ 0.03). Thus, the results indicate an additive effect of HBP and resistin in induction of inflammatory responses.

We also assessed IL-8 in the sepsis cohorts, and, as expected, elevated levels were detected in all cohorts (median ng/ml (range); STSS: 626 (306–1985); Gram-positive sepsis: 225 (28–1611); Gram-negative sepsis: 118 (51–2118)). It was noteworthy, that in STSS patients a strong correlation between IL-8 levels and HBP (r = 0.84, p = 0.009), as well as IL-8 and resistin (r = 0.86, p = 0.006) was observed, whereas no such correlation was evident in the other sepsis cohorts.

### Release of high amounts of both HBP and resistin follows neutrophil activation induced by GAS, but not S. aureus or E. coli

**Figure 3:**
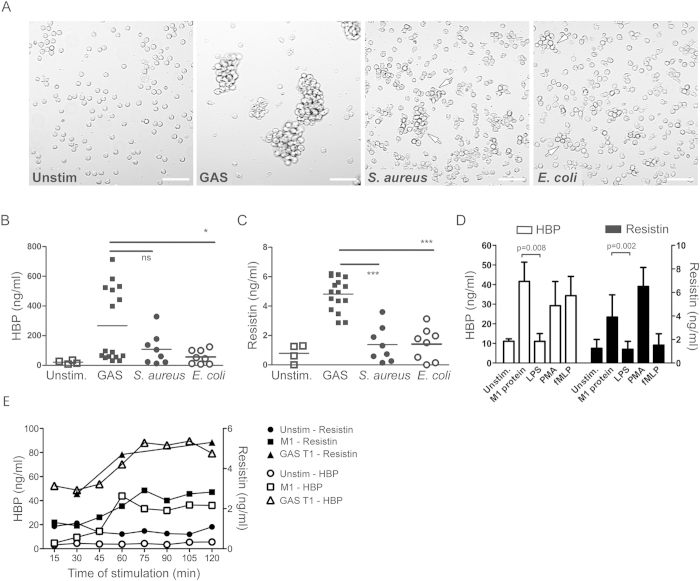
Release of HBP and resistin differs depending on bacterial stimuli. Primary neutrophils isolated from blood of healthy donors were stimulated for 2 hours with either fixed bacteria or different bacterial components. HBP and resistin levels were determined in cell culture supernatants by ELISA. (**A**) Microscopic bright field images of neutrophils stimulated with the indicated bacteria (20 cfu/cell). Arrows indicate aggregated neutrophils. The scale bars indicate 100 μm. (**B,C**) HBP and resistin levels in supernatants from cells stimulated with GAS (n = 4), *S. aureus* (n = 2) and *E. coli* (n = 2) (67 cfu/cell). The data show results from four experiments using different donors. Kruskal Wallis with Dunns test was used for comparison between groups. (**D**) HBP and resistin levels in supernatants of neutrophils stimulated with the streptococcal M1-protein (0.5 μg/ml), LPS (50 ng/ml), and the known neutrophil activators PMA (25 ng/ml) and fMLP (5 μg/ml). Mean ± SD of results from five separate experiments using cells from different donors. Mann-Whitney *U* test was used for comparison between and differences are shown in *p* values, *P < 0.05, ***P < 0.001. (**E**) Kinetics of HBP and resistin levels in supernatants of neutrophils stimulated with fixed GAS T1 strain (67 cfu/cell) or with streptococcal M1-protein (1 μg/ml) for 2 hours. The figure shows one representative experiment out of two performed.

As the clinical data indicated that the neutrophil response varied depending on the infectious agent, we compared the ability of different bacterial stimuli to trigger neutrophil activation and degranulation. To this end, primary human neutrophils isolated from healthy blood donors were exposed to different clinical septic shock isolates, namely *E. coli*, *S. aureus* and GAS. Filtered bacterial supernatants as well as fixed bacteria prepared from overnight cultures were used to stimulate neutrophils for 2 hours, after which HBP and resistin were measured in cell culture supernatants. Visualization of neutrophils exposed to fixed bacteria revealed starkly different responses with GAS resulting in an almost complete aggregation of the cells, while *S. aureus* some, and *E. coli* only marginal aggregation (Fig. 3A). Similarly, high levels of HBP and resistin were detected after stimulation with fixed GAS strains (n = 4), whereas markedly lower levels were triggered by fixed *S. aureus* (n = 2) or *E. coli* strains (n = 2) (Fig. 3B,C). Stimulation with bacterial supernatants (1:50 and 1:500 dilutions) failed to induce either HBP or resistin release. Neutrophils were also stimulated with purified bacterial proteins, including the streptococcal M1-protein and the endotoxin LPS. In agreement with the bacterial stimulation experiments, the streptococcal M1-protein resulted in release of significantly higher amounts of both HBP and resistin, as compared to LPS that was a weak trigger of release of either mediator (p < 0.008) (Fig. 3D). A kinetic experiment revealed a similar profile for HBP and resistin release in response to M1-protein and fixed GAS with both factors starting to appear after 15–30 minutes depending on stimuli (Fig. 3E).

**Figure 4:**
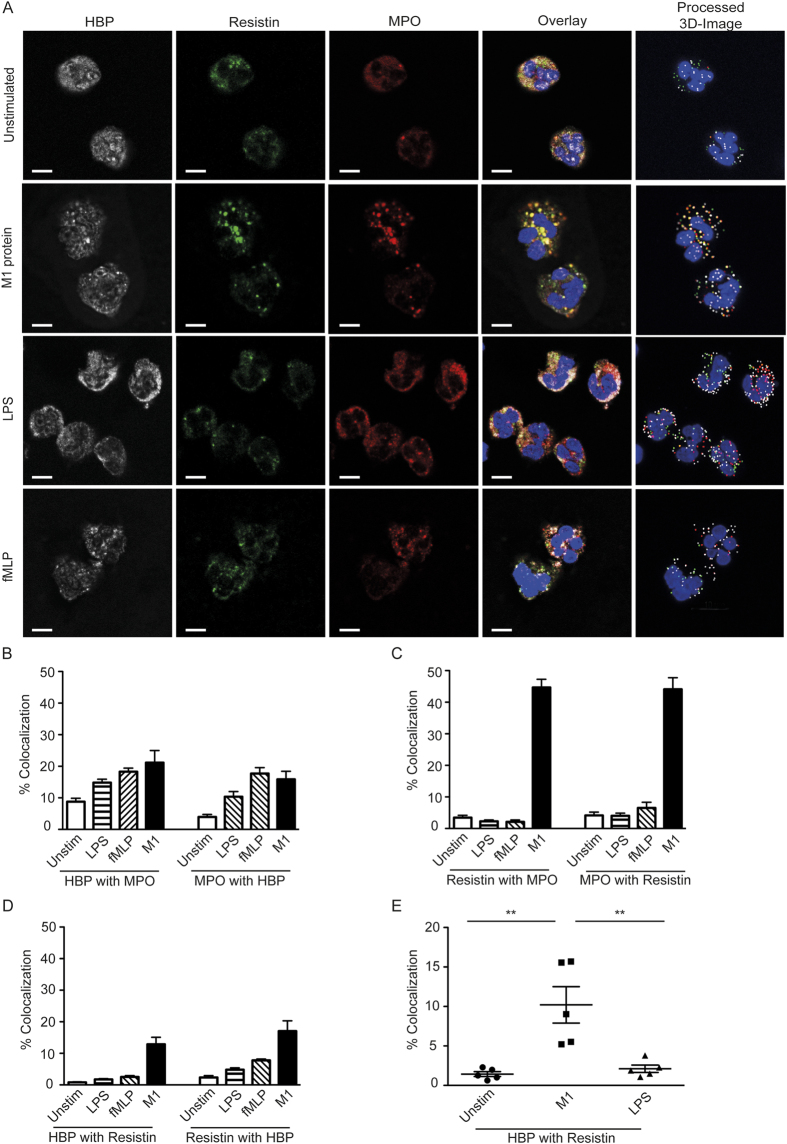
Subcellular localization of HBP and resistin in neutrophils. Neutrophils from healthy donors were isolated and stimulated with LPS (50 ng/ml), fMLP (5 μg/ml) or streptococcal M1-protein (1 μg/ml) for 2 h. The neutrophils were immunoflourescently stained for HBP (white), resistin (green) and MPO (red), the cell nuclei were visualized using DAPI (blue). Using scanning laser confocal microscopy, Z-stacks of the cells were acquired and vesicles positive for HBP, resistin or MPO identified using the Imaris image analysis software (processed 3D-Image). The degree of colocalization between the above-mentioned markers in the processed images was then quantified using Imaris analysis software. (**A**) Shows images of cells from a high responder. Bars indicate 5 μm. (**B–D**) Percentage of co-localization between factors as indicated. (**E**) Differences in co-localization in neutrophils unstimulated or stimulated with either M1-protein or LPS. Statistical significant differences were determined using the Mann Whitney test, **P < 0.01. Data represent results obtained from multiple field analyses of two experiments using different donors.

Thus, the data demonstrates specific neutrophil responses to different stimuli and a seemingly synchronized release of HBP and resistin after streptococcal induced neutrophil activation. It was therefore of interest to analyse granule mobilization. For this purpose, we compared unstimulated and stimulated neutrophils for intracellular HBP, resistin and the azurophilic marker MPO through triple immunoflourescent stainings combined with confocal microscopy (Fig. 4A). In unstimulated neutrophils, numerous positive granules for each factor were detected. A large portion of HBP and resistin positive granules also contained MPO, confirming their presence in azurophilic granules. However, only few granules were double positive for both HBP and resistin (Fig. 4A, unstimulated control). In contrast, neutrophils activated with different stimuli displayed starkly different granule staining patterns depending on stimuli (Fig. 4). Streptococcal M1-protein stimulated cells displayed a greater co-localization of resistin and MPO as well as HBP and resistin, when compared to unstimulated cells (Fig. 4). Quantification of the degree of co-localization between HBP and resistin showed a significantly higher increase in cells stimulated with M1-protein as compared to LPS stimulated cells (Fig. 4D,E). Thus, the data concurs with the noted differences in *in vitro* responses elicited by various bacterial stimuli.

### Streptococcal activation of neutrophils involves multiple surface associated proteins

**Figure 5:**
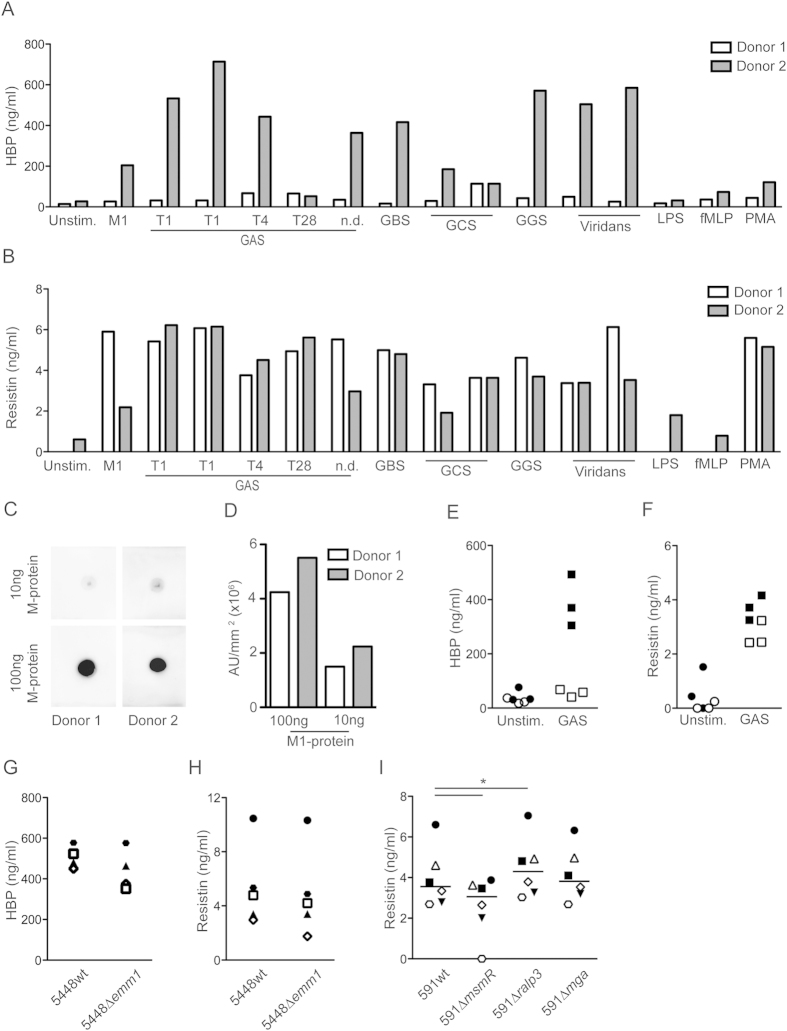
Pronounced neutrophil stimulatory effect can be seen across streptococcal species and involves multiple surface proteins. (**A**,**B**) HBP and resistin levels in supernatants from neutrophils isolated from two donors (D1 and D2) stimulated with different fixed streptococcal species (67 cfu/cell), as indicated in the figure. ND denotes serotype not determined. (**C**) Plasma from the high- and low responding donor (D1 and D2) was tested for anti-M1 protein antibodies by dot blot analyses. Purified M1-protein (100 and 10 ng) was spotted on to a PVDF-membrane, after which donor plasma was added and incubated over night. Bound anti-M1 antibodies were detected using HRP coupled anti-human IgG. (**D**) The intensity of positive stain was quantified and plotted as arbitrary units (AU)/mm^2^. (**E**,**F**) HBP and resistin levels in culture supernatants from neutrophils, either unstimulated (Unstim.) or stimulated with fixed GAS T1 (GAS), in the presence (filled symbols) or absence (open symbols) of 10% autologous plasma. The experiment was done using cells from three different donors. (**G**,**H**) HBP and Resistin levels in culture supernatants from neutrophils stimulated with fixed wild type or corresponding isogenic deletion mutants of the *emm1* gene. Shown are the results from 4–5 experiments using different donors, indicated by specific symbols. (**I**) Resistin levels in culture supernatants from neutrophils stimulated with fixed wild type or corresponding isogenic deletion mutants of the indicated genes. Results were obtained from five experiments using different donors, the respective donors are indicated by specific symbols and the line indicates median. Wilcoxon matched-pairs signed rank test was used for comparison between groups, *P < 0.05.

To further explore whether this neutrophil-stimulatory effect is exclusive to GAS or shared by other streptococcal species, we included group B, C and G streptococcus (GBS, GCS, and GGS), as well as viridans group streptococcal strains collected from septic shock patients in addition to five GAS strains including strains of serotypes T1, T4 and T28, as well as one strain of unknown type. All strains were found to induce a strong resistin response in the two donors tested, whereas they only elicited HBP release in one of the donors (Fig. 5A,B). This inter-individual variation with low or high responders was seen particularly for streptococcal triggered HBP release (Fig. 5A,B). Donor variation in streptococcal M1-protein triggered HBP release has previously been reported, and found to be linked to the presence of high anti-M1 protein specific antibody titres in the high responder^21^. In line with this report, dot blot analyses of anti-M1 protein antibodies in plasma of the high and low responder revealed a stronger signal when plasma of the higher responder (donor 2) was used as compared to the low responder (donor 1) (Fig. 5C,D).

Furthermore, the presence of other plasma proteins, in particular fibrinogen, has also been implicated as an important factor for streptococcal M1-protein triggered HBP release^18^,^21^. In accordance with these reports, stimulation experiments carried out in the presence or absence of 10% autologous plasma showed a plasma-dependency for fixed GAS triggered HBP release (Fig. 5E). Notably, this dependency was only noted for HBP and resistin release was only marginally reduced when plasma was absent (Fig. 5F), suggesting different signal requirements for the release of these two factors.

The finding that GBS, GCS, GGS, and viridans group streptococci activated neutrophils to a similar extent as GAS is of interest as they share several surface attached factors such as M- or M-like proteins, pili, as well as fibronectin- and collagen-binding proteins (reviewed in^22^). In an attempt to identify the stimulatory factors, we first explored the role of surface-attached M1 protein by use of an M1-protein deficient mutant of 5448. However, the results showed only a marginal, non-significant, reduction in HBP and resistin release when neutrophils were stimulated with the mutant strain (Fig. 5G,H); thus implicating also other surface-attached factors and possible involvement of multiple factors in neutrophil activation. To explore this, we exploited a set of GAS strains deficient in the gene regulatory systems including Mga which is positive regulator of *emm*- and *emm*-like gene expression within the Mga-locus^23^, as well as MsmR and Ralp3, both of which influence the FCT region encoding for fibronectin- and collagen-binding proteins as well as the T-pilus^24^,^25^. Similarly to the 5448Δ*emm*1, the 591Δ*mga* mutant induced equal response as the wild type strain (Fig. 5I). In contrast, 591Δ*msm*R triggered resistin release, but at a significantly reduced level as compared to the wild type strain, while 591Δ*ralp*3 triggered an increased response (Fig. 5I). Taken together, the results implicate a multifactorial activation of neutrophils involving surface attached proteins encoded by the FCT-locus, rather than M- and M-like proteins, as triggers for neutrophil activation.

**Figure 6:**
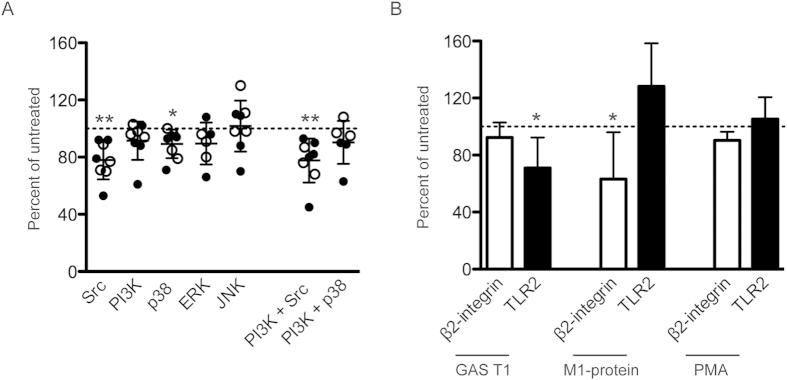
Signalling molecules and receptors involved in the release of resistin. Resistin levels in cell culture supernatants of neutrophils from healthy donors, pre-treated for 30 min with inhibitors targeting the signalling molecules Src (PP1), PI3K (LY294002), MAPK p38 (SB203580), ERK (PD98059), and JNK (SP600125) (**A**) or antibodies against β2-integrins (anti-human CD18) and TLR-2 (anti-Human CD282) (**B**), and stimulated with fixed GAS for 2 h. In A, open and filled symbols show 5448 and the T1 isolate, respectively. Mean ± SD of results from 3–9 experiments using different donors presented as per cent relative to untreated cells, the dashed line indicates 100 per cent. The inhibitors did not influence the cell response, as unstimulated cells had similar background levels in the presence or absence of the inhibitors. Wilcoxon matched-pairs signed rank test was used for comparison between groups, *P > 0.05,**P < 0.01.

Finally, we explored host signals and putative receptors involved in the neutrophil activation. For this purpose, neutrophils were pre-incubated with inhibitors targeting Src-family kinases, phosphatidylinositol 3-kinases (PI3K), and the MAPK p38, extracellular signal-regulated kinase (ERK 1/2), and c-Jun N-terminal kinase (JNK), followed by stimulation with fixed GAS strains. A consistent reduction in the resistin release elicited by GAS strains was noted in neutrophils pre-treated with either Src-family kinase or p38 inhibitors, as compared to the untreated control (p > 0.05) (Fig. 6A). A small effect, although non-significant, was also seen with an inhibitor targeting PI3K, whereas inhibition of ERK1/2 and JNK had no effect (Fig. 6A). No further reduction in response was achieved through inhibition of PI3K + Src or PI3K + p38 (Fig. 6A). We also tested whether β2-integrin or TLR2 were involved in these responses by use of blocking antibodies. As previously reported^18^ blocking of β2-integrin resulted in significant reduction of streptococcal M1-protein, but not fixed GAS, elicited responses (Fig. 6B). In contrast, the reverse was true when TLR2 was blocked, with a reduction in response to fixed GAS (Fig. 6B). Responses elicited by PMA were not affected by either antibody. It should be noted that the inhibition was far from complete, which is in line with a multifactorial activation involving several receptors and signals.

## Discussion

In this study we demonstrate that there is a marked variation in neutrophil responses towards different bacterial pathogens. Human primary neutrophils were exposed to different clinical strains isolated from severe sepsis/septic shock cases and degranulation was assessed by measurement of the two sepsis-associated factors HBP and resistin. The stimulation experiments revealed that streptococcal strains, including not only GAS, but also GBS, GCS, GGS and viridans group streptococci as well as streptococcal M1-protein were potent triggers of both HBP and resistin release, whereas *S. aureus*, *E. coli* and LPS were all poor inducers. Although LPS is a poor inducer in comparison to the streptococcal strains, it should be noted that the magnitude of resistin release is equal to that reported in other studies^26^. Furthermore, this relatively poor effect of the TLR-agonist LPS is in agreement with previous reports showing that, although LPS has a priming effect, it is a weak agonist of neutrophil exocytosis^27^.

Moreover, supernatants prepared from the bacterial cultures failed to induce any HBP or resistin release. Thus, in the case of streptococcal bacterial activation, our data suggest that surface-associated factors, rather than secreted factors, are involved in triggering neutrophil degranulation. However it should be noted that in a previous report, HBP-release was observed following stimulation with GAS overnight culture supernatants, but only by supernatants containing high levels of streptolysin O (SLO)^28^. Thus we cannot exclude that the lack of a response induced by supernatants might be due to a low production of SLO by the strains used in this study. This also raises the question as to how the expression of virulence factors *in vitro* compares to that in patients. Humoral responses in patients show that superantigens and streptolysins are produced, but there are no data pertaining to their concentrations. Here we are using supernatants containing a mixture of the secreted virulence factors, as this is as close to the clinical setting as we can technically reproduce. Along the same lines, live infection is clinically relevant; however, in this study fixed bacteria were deemed necessary in order to be able to compare bacterial factors triggering neutrophil activation. Live infection would be associated with confounders such as varying degree of phagocytosis, intracellular replication, as well as toxin-mediated cytotoxicity, which would complicate interpretation.

Previous reports on neutrophil activation by streptococcal strains have focused on GAS and particularly on the streptococcal M-protein, which in a soluble form was identified as a key trigger of neutrophil activation and degranulation^18^. Herwald *et al.*^18^ delineated the underlying mechanism and showed that it entailed complex formation with fibrinogen and subsequent β2-integrin binding. In this study, strains of different types, including T1 (coupled to *emm*1/M1 serotype) as well as T4 and T28 types were tested. The latter two types both express the M-related protein MRP4 that, similarly to M1-protein, has two fibrinogen binding sites^29^. Streptococcal strains commonly express fibrinogen-binding proteins, which in GAS, GBS, GCS and GGS predominantly consist of members of the M/M-like protein family^22^. In viridans group streptococci, a phage lysin with fibrinogen-binding capacity has been identified^30^. Hence, streptococcal surface associated M-protein and other fibrinogen-binding proteins represented putative candidates that might contribute to the noted neutrophil activation. However, experiments using an M1-deficient GAS strain yielded only a marginal reduction in the response; thus suggesting that surface attached M-protein is not the main trigger or that there are multiple streptococcal factors involved. Streptococcal surface attached factors including M- and M-like proteins, pili structures and extracellular matrix binding proteins, such as Fibronectin and Collagen binding proteins are under the control of gene regulatory systems. We therefore tested bacterial mutants deficient in gene regulatory systems including Mga, which is a regulator influencing *emm* and *emm*-like genes, as well as MsmR and Ralp3 influencing the FCT-locus encoding for Fibronectin- and Collagen-binding proteins as well as T-pilus. Similar to the M1-mutant strain, deletion of Mga did not impair the stimulatory capacity. Furthermore, the decreased Mga-operon transcript abundance and increased FCT-locus transcription in Ralp3 knockout strain, underlines rather the involvement of FCT-region proteins than *emm* and *emm*-like genes in neutrophil activation. In addition, the MsmR-deficient strain triggered a reduced response. In *msm*R- and *ralp*3-mutants several genes are differentially expressed including the genes encoding Fibronectin binding protein F2 (*prt*F2) and Collagen binding protein (*cpa*). *prt*F2 and *cpa* were both down regulated in the *msm*R-mutant while upregulated in the *ralp*3-mutant^24^,^25^. Although the stimulatory capacity was reduced with the *msm*R-mutant, suggesting involvement of the FCT-region proteins as neutrophil activators, it still triggered a substantial activation. Taken together, this implies the involvement of multiple factors in streptococcal triggered neutrophil activation. Also attempts to block signal-transduction pathways using pharmacological inhibitors and blocking antibodies revealed a role for Src family kinases and TLR2 in streptococcal neutrophil activation and degranulation; however, the blocking effect was far from complete, which is expected if several bacterial ligands are involved and interact with multiple receptors.

The study revealed marked inter-individual variations particularly in HBP-responses between donors with some being low responders and others high responders. The HBP, but not resistin, response was fully dependent on the presence of plasma, and in accordance with a previous report, the donor variation in HBP-release upon GAS stimulation seemed to be linked to presence or absence of anti-M protein antibodies^21^. In the report by Kahn *et al.* it was proposed that the high response was related to presence of antibodies targeting the central region of the M-protein resulting in complexes containing M-protein, fibrinogen and IgG, which triggered HBP release through dual engagement of Fc-receptor and β2-integrin^21^. In our study, the high responder did not only respond strongly to M1 protein and GAS strains but also to GBS, GCS, GGS and viridans group streptococci. At present, the underlying mechanism for this general high response to streptococcal strains remains to be elucidated, but as M and M-like proteins share homologies in the central regions, it is tempting to speculate that the effect might be related to cross-reactive antibodies. Notably, streptococcal strains triggered release of resistin in all donors and the donor variation was exclusively linked to HBP release; thus, indicating involvement of different receptors and/or signal-transducing pathways for exocytosis of HBP and resistin.

Visualization of the granule content through intracellular immunostainings of stimulated cells followed by confocal microscopy revealed that HBP and resistin were localized predominantly in separate granules, many of which were MPO-positive azurophilic granules. Notably, upon activation a pronounced increase in co-localization of HBP and resistin was evident in cells stimulated with streptococcal M1-protein, but not with LPS nor with fMLP. In addition, the granules appeared larger in size, which indicates a mobilization of HBP- and resistin-positive granules in response to M1-protein. Taken together with the similar kinetic response profile, the data is indicative of a synchronized release of these two factors in response to streptococcal stimulation. This was further supported by analyses of HBP and resistin responses in septic shock cohorts. All septic shock patients had elevated levels of plasma HBP and resistin as compared to critically ill non-infectious patients, and a positive correlation between plasma HBP and resistin levels was found in all patient cohorts (i.e. Gram-positive and Gram-infections, STSS caused by GAS. However, the correlation was particularly striking in the STSS cohort, both systemically in plasma as well in soft tissue biopsies. As it had not previously been explored how the co-presence of these two effector molecules might influence inflammation, we also tested the response of PBMC to HBP and resistin alone or in combination. The results demonstrated that cells exposed to both factors in combination responded with a significantly higher inflammatory response, as determined by IL-8 release. Notably, measurement of IL-8 levels in the patients revealed a positive correlation between IL8 with HBP and resistin, respectively, in STSS patients, but not in the large sepsis cohort.

Taken together, the data support the concept that neutrophil activation differs depending on the bacterial stimuli, which may have clinical implications. Considering the greater stimulatory capacity of the streptococcal strains tested in this study, it seems plausible that neutrophil activation and degranulation may play a central role in the pathology of streptococcal infections. This has important implications for therapeutic strategies, especially considering that several trials have attempted to enhance neutrophil recruitment and function in sepsis. On this note, our data underline the importance of further studies on this topic to provide insight into patient groups most likely to benefit from neutrophil modulation.

## Experimental procedures

### Patient cohorts

Plasma samples from culture-positive severe sepsis and septic shock patients (n = 88), including 20 and 28 patients with confirmed Gram-positive and Gram-negative bacterial infections, respectively, and a reference group of non-infected critically ill patients (n = 31) enrolled at Karolinska University Hospital Huddinge were used^19^. The three cohorts were well matched with respect to age, gender and severity of infection based on APACHE II score at the day of inclusion (median APACHE II (range) Gram-positive: 23 (12–37); Gram-negative: 25 (9–45); reference group: 22 (9–33)). Plasma from patients with STSS caused by GAS (n = 8) were provided from a sepsis study conducted at Lund University Hospital^11^. No APACHE II score was available from the STSS-cohort. All plasma samples were collected during the acute septic episode.

Snapfrozen tissue biopsies from patients with necrotizing fasciitis or severe cellulitis caused by GAS (n = 9) were used. This material has been described previously^31^,^32^.

All studies were ethically approved by Karolinska University Hospital, the University of Toronto, and Lund University Hospital, and all experiments were carried out in accordance with the approved guidelines. Written informed consent was obtained from all patients or their legal guardians.

### Bacterial isolates and factors

Blood isolates, including GAS (n = 4), *E. coli* (n = 2) and *S. aureus* (n = 2), collected from the septic shock patients in respective cohort described above were used. The GAS strains were of serotypes T1 (n = 2), T4 and T28. Also, streptococcal strains, including group B (GBS; n = 1), group C (GCS; n = 2), group G (GGS, n = 1), and viridans group streptococci (n = 2), isolated from patients with severe sepsis were used. In addition, isogenic mutants deficient in M1-protein (5448Δ*emm*1)^33^ or in gene regulatory systems including 591Δ*msmR*^25^, 591Δ*ralp3*^24^ and 591Δ*mga*^23^ were used. The streptococcal strains were cultured overnight (16 h) in Todd-Hewitt medium with 1.5% yeast, whereas LB medium was used for *E. coli* strains and CCY medium for *S. aureus*. Different media were used to obtain optimal growth conditions known to support exotoxin production for respective bacteria. Media controls were included in all stimulation experiments. Following centrifugation, the supernatants were sterile-filtrated and the bacterial pellet fixed with 1% paraformaldehyde for 45 minutes.

The streptococcal M1-protein was purified from the AP1-derived isogenic mutant MC25 strain as previously described^34^. Commercially available lipopolysaccharide (LPS) from *E. coli* (Sigma-Aldrich, St Louis, MO) was used.

### Antibacterial activity assay

*S. pyogenes* (T1 strain), *S. aureus* and *E. coli* were grown in liquid broth at 37 °C over night. The cultures were diluted 1:50 (*S. pyogenes*) and 1:180 for *E. coli* and *S. aureus*. In order to determine the antibacterial effect of HBP (R&D systems, Minneapolis, MN) and resistin (PeproTech, Rocky Hill, NJ), the bacterial cultures were exposed to different concentrations of the peptides. Final concentrations of 5 μg/ml, 2 μg/ml, 1 μg/ml and 0.1 μg/ml of HBP and 2 μg/ml, 1 μg/ml, 0.1 μg/ml of resistin (Innovagen, Lund Sweden) were tested. An optical density (OD) curve was generated based on the turbidity measurement at 420–580 mm, using a Bioscreen C over a time of 24 h. Measurements were made every 15 min and data exported to a PC. Data were analysed with GraphPad Prism version 6.0 for Mac (GraphPad software).

### Infection and stimulation of human primary cells

Human neutrophils and peripheral blood mononuclear cells (PBMCs) were isolated from venous blood collected from healthy individuals by Polymorphprep or Ficoll-Hypaque (Axis-Shield, Oslo, Norway) gradient centrifugation, respectively. Cells were resuspended in RPMI-1640 supplemented with 5% FCS, 10 mM L-glutamine, Penicillin (100 U/ml)/Streptomycin (100 μg/ml) and 25 mmol/L HEPES (all from Thermo Scientific, Hyclone, South Logan, UT). 5 × 10^5^ neutrophils/ml were stimulated with streptococcal M1-protein, LPS (extracted from *E. coli*), Phorbol Myristate Acetate (PMA) (Sigma-Aldrich, St Louis, MO), N-formyl-methionine-leucine-phenylalanine (fMLP) (Calbiochem, San Diego, CA), or with fixed bacteria (67 cfu/cell, unless otherwise indicated) or bacterial supernatants (1:50 and 1:500 dilutions). In case of inhibition of molecules or receptors, isolated neutrophils were pre-incubated with 10 μM of inhibitor or 10 μg blocking antibody for 30 minutes.

The dose of fixed bacteria was chosen based on dose-response experiments, which showed a stable response over a broad multiplicity of infection (MOI) range, i.e. 17–260 cfu/cell. Also the concentrations of streptococcal M1-protein and LPS were chosen based on dose-response experiments in which 0.5 or 1 μg/ml gave a peak response for M1 stimulation, whereas LPS showed an equal response over the range of 50 ng/ml–1 μg/ml. Streptococcal M1-protein, bacterial supernatants and fixed bacteria were pre-incubated for 10 minutes at 37 °C with 10% autologous plasma to allow for fibrinogen-complex formation as described previously^18^. At defined time points, the cells were centrifuged and cell-free culture supernatants collected for analyses.

PBMCs (1 × 10^6 ^cells/ml) were stimulated with HBP (R&D systems, Minneapolis, MN) and resistin (PeproTech, Rocky Hill, NJ), each factor alone and in combination for 2 h after which culture supernatants were collected.

The following blocking antibodies and pharmacological inhibitors were used: anti-β2 integrin (anti-human CD18, clone IB4, Merk Millipore, Billerica, MA, USA), anti-TLR2 (anti-human CD282, clone TL2.1, eBioscience, San Diego, CA, USA), LY294002 (PI3K), PD98059 (ERK1/2), SB203580 (MAPK p38), PP1 (Src-family kinases) and SP600125 (JNK) (all from Sigma-Aldrich, St Louis, MO, USA). These particular molecules were chosen as protein phosphorylation is a critical step in neutrophil activation, and the Src-family of tyrosine kinases has been implicated in the control of granule exocytosis in human neutrophils^35^,^36^,^37^.

### Dot-blot assay

Streptococcal M1-protein (obtained as described above) or PBS (negative control) was spotted onto a PVDF-membrane, and left to absorb for 30 min. The M1-probed membrane was subsequently washed using a 0.1% Tween (Sigma-Aldrich, St Louis, MO) in PBS solution (PBST), and blocked for 2 h using 5% milk in PBST. After blocking, the membrane was washed using PBST and incubated with donor plasma (diluted 1:10 with 2.5% milk in PBST) over night at 4 ^o^C. Following the over night incubation, the membrane was carefully washed and incubated with a horseradish peroxidase labelled secondary anti-Human IgG antibody (GE Healthcare, Piscataway, NJ) (diluted 1:10.000 with 2.5% milk in PBST). The blot was developed using an electrochemiluminescence kit (Thermo Scientific, South Logan, UT). As a positive control, the membrane was incubated with anti-M1 rabbit serum and detected using anti-rabbit IgG. Intensities were evaluated using a luminescent image analyser (LAS-4000 mini) and quantified using the Multi Gauge software version 3.2 (both from Fujifilm, Minato, Tokyo, Japan).

### Analysis of secreted HBP, resistin and IL-8

HBP was analysed by ELISA as previously described^8^. Resistin was analysed by a commercially available ELISA (BioVendor, Brno, Czech Republic), according to the manufacturer’s instructions. IL-8 levels were measured in a Luminex analysis (Invitrogen, Grand Islands, NY).

### Immunostainings of tissue biopsies and primary human cells

Snapfrozen biopsies (n = 9) were sectioned, fixed, immunostained, and analysed as described previously^15^. The whole section was analysed by acquired computerized image analysis (ACIA). The results are presented as ACIA values, which equals the percentage of the positively stained area x the mean intensity of positive staining.

Stimulated neutrophils were fixed with 2% paraformaldehyde for 10 min, washed with PBS and added to glass coverslips using cytospin. Triple immunofluorescence stainings were conducted as follows; the cells were permeabilized using 0.1% saponin in PBS, and initially blocked with 2% FCS in PBS-saponin for 5 minutes at 37 °C. This was followed by 30 minutes blocking with 0.1% BSA-c (Sigma-Aldrich, St Louis, MO) in PBS-saponin, after which the primary antibodies were added for 30 minutes at 37 °C. Prior to addition of the fluorophore-conjugated secondary antibodies, cells were blocked for 30 min with 1% normal goat serum in PBS-saponin. The secondary antibodies were incubated for 30 min in the dark.

The following antibodies were used: anti-neutrophil elastase (NP57); anti-CD68 (EBM11) (both from DakoCytomation, Carpinteria, CA), anti-Resistin (AF1359 and MAB1359)(both from R&D Systems, Minneapolis, MN), anti-MPO (sc-33595, Santa Cruz, Dallas, Texas), and the secondary antibodies donkey anti-mouse IgG-Alexa488 (1226927), donkey anti-rabbit IgG-Alexa546 (1182675) and donkey anti-goat IgG-Alexa647 (1235825) (all from Molecular Probes, Grand Islands, NY). HBP was identified with purified IgG from a polyclonal anti-HBP rabbit serum or IgG purified from a monoclonal anti-HBP mouse serum (2F23A)^38^.

For evaluation a Nikon A1R confocal microscope was used (Nikon Instruments, Amstelveen, the Netherlands). Z-stacks were acquired for 3–5 fields of view, using a 100× oil plan apochromatic objective.

### Co-localization analysis

Z-stack images were analysed using the Imaris 3-D image analysis version 7.6.3 (Bitplane, Zurich, Switzerland). The Imaris cell function was then applied to automatically locate the vesicles inside the cells based on size and intensity thresholds defined by the user. Measurement of co-localization was carried out by masking each respective vesicle type, creating new channels for each marker. Total number of co-localized voxels was measured, and thresholds were automatically computed by using orthogonal regression analysis of the image’s scatterplots in combination with the Pearson’s coefficient approach.

### Statistical evaluation

Data were analysed by GraphPad Prism version 4.0 for Windows (GraphPad software). Mann-Whitney *U* test, Wilcoxon matched-pairs signed rank test or Kruskal Wallis with Dunns test was used for comparison between groups. Correlations between variables were determined by use of Pearson correlation test or, in the case of non-Gaussian distribution of the data, Spearman rank correlation coefficient. Differences were considered significant when *p* < 0.05.

## Additional Information

**How to cite this article**: Snäll, J. *et al.* Differential neutrophil responses to bacterial stimuli: Streptococcal strains are potent inducers of heparin-binding protein and resistin-release. *Sci. Rep.* **6**, 21288; doi: 10.1038/srep21288 (2016).

## References

[b1] Weiss S. J. Tissue destruction by neutrophils. N Engl J Med 320, 365–376, 10.1056/NEJM198902093200606 (1989). 2536474

[b2] Kovach M. A. & Standiford T. J. The function of neutrophils in sepsis. Curr Opin Infect Dis 25, 321–327, 10.1097/QCO.0b013e3283528c9b (2012). 22421753

[b3] Kipnis E. Neutrophils in sepsis: battle of the bands. Crit Care Med 41, 925–926, 10.1097/CCM.0b013e31828042d8 (2013). 23425832

[b4] Brown K. A. *et al.* Neutrophils in development of multiple organ failure in sepsis. Lancet 368, 157–169, 10.1016/S0140-6736(06)69005-3 (2006). 16829300

[b5] Drifte G., Dunn-Siegrist I., Tissieres P. & Pugin J. Innate immune functions of immature neutrophils in patients with sepsis and severe systemic inflammatory response syndrome. Crit Care Med 41, 820–832, 10.1097/CCM.0b013e318274647d (2013). 23348516

[b6] Sundén-Cullberg J. *et al.* Pronounced elevation of resistin correlates with severity of disease in severe sepsis and septic shock. Crit Care Med 35, 1536–1542 (2007). 17452927 10.1097/01.CCM.0000266536.14736.03

[b7] Linder A. *et al.* Elevated plasma levels of heparin-binding protein in intensive care unit patients with severe sepsis and septic shock. Crit Care 16, R90, 10.1186/cc11353 (2012). 22613179 PMC3580636

[b8] Tapper H., Karlsson A., Mörgelin M., Flodgaard H. & Herwald H. Secretion of heparin-binding protein from human neutrophils is determined by its localization in azurophilic granules and secretory vesicles. Blood 99, 1785–1793 (2002). 11861296 10.1182/blood.v99.5.1785

[b9] Soehnlein O. & Lindbom L. Neutrophil-derived azurocidin alarms the immune system. J Leukoc Biol 85, 344–351, 10.1189/jlb.0808495 (2009). 18955543

[b10] Gautam N. *et al.* Heparin-binding protein (HBP/CAP37): a missing link in neutrophil-evoked alteration of vascular permeability. Nat Med 7, 1123–1127, 10.1038/nm1001-1123 (2001). 11590435

[b11] Linder A., Christensson B., Herwald H., Björck L. & Akesson P. Heparin-binding protein: an early marker of circulatory failure in sepsis. Clin Infect Dis 49, 1044–1055 (2009). 19725785 10.1086/605563

[b12] Lehrke M. *et al.* An inflammatory cascade leading to hyperresistinemia in humans. PLoS Med 1, e45, 10.1371/journal.pmed.0010045 (2004). 15578112 PMC529430

[b13] Nagaev I., Bokarewa M., Tarkowski A. & Smith U. Human resistin is a systemic immune-derived proinflammatory cytokine targeting both leukocytes and adipocytes. PLoS One 1, e31, 10.1371/journal.pone.0000031 (2006). 17183659 PMC1762367

[b14] Bokarewa M., Nagaev I., Dahlberg L., Smith U. & Tarkowski A. Resistin, an adipokine with potent proinflammatory properties. J Immunol 174, 5789–5795 (2005). 15843582 10.4049/jimmunol.174.9.5789

[b15] Johansson L. *et al.* Neutrophil-derived hyperresistinemia in severe acute streptococcal infections. J Immunol 183, 4047–4054, 10.4049/jimmunol.0901541 (2009). 19717514

[b16] Kobayashi S. D. *et al.* Bacterial pathogens modulate an apoptosis differentiation program in human neutrophils. Proc Natl Acad Sci USA 100, 10948–10953, 10.1073/pnas.1833375100 (2003). 12960399 PMC196908

[b17] Soehnlein O. *et al.* Neutrophil degranulation mediates severe lung damage triggered by streptococcal M1 protein. Eur Respir J 32, 405–412, 10.1183/09031936.00173207 (2008). 18321926

[b18] Herwald H. *et al.* M protein, a classical bacterial virulence determinant, forms complexes with fibrinogen that induce vascular leakage. Cell 116, 367–379 (2004). 15016372 10.1016/s0092-8674(04)00057-1

[b19] Linnér A. *et al.* Short- and long-term mortality in severe sepsis/septic shock in a setting with low antibiotic resistance: a prospective observational study in a Swedish university hospital. Front Public Health 1, 51, 10.3389/fpubh.2013.00051 (2013). 24350220 PMC3859970

[b20] Påhlman L. I. *et al.* Streptococcal M protein: a multipotent and powerful inducer of inflammation. J Immunol 177, 1221–1228 (2006). 16818781 10.4049/jimmunol.177.2.1221

[b21] Kahn F. *et al.* Antibodies against a surface protein of *Streptococcus pyogenes* promote a pathological inflammatory response. PLoS Pathog 4, e1000149, 10.1371/journal.ppat.1000149 (2008). 18787689 PMC2522270

[b22] Nobbs A. H., Lamont R. J. & Jenkinson H. F. Streptococcus adherence and colonization. Microbiol Mol Biol Rev 73, 407–450, 10.1128/MMBR.00014-09 (2009). 19721085 PMC2738137

[b23] Fiedler T. *et al.* Impact of the *Streptococcus pyogenes* Mga regulator on human matrix protein binding and interaction with eukaryotic cells. International journal of medical microbiology : IJMM 300, 248–258, 10.1016/j.ijmm.2009.07.004 (2010). 20097132

[b24] Siemens N. *et al.* Effects of the ERES pathogenicity region regulator Ralp3 on *Streptococcus pyogenes* serotype M49 virulence factor expression. Journal of bacteriology 194, 3618–3626, 10.1128/jb.00227-12 (2012). 22544273 PMC3393516

[b25] Nakata M., Podbielski A. & Kreikemeyer B. MsmR, a specific positive regulator of the *Streptococcus pyogenes* FCT pathogenicity region and cytolysin-mediated translocation system genes. Molecular microbiology 57, 786–803, 10.1111/j.1365-2958.2005.04730.x (2005). 16045622

[b26] Furugen R., Hayashida H. & Saito T. Porphyromonas gingivalis and Escherichia coli lipopolysaccharide causes resistin release from neutrophils. Oral Dis 19, 479–483, 10.1111/odi.12027 (2013). 23083402

[b27] Brzezinska A. A. *et al.* The Rab27a effectors JFC1/Slp1 and Munc13-4 regulate exocytosis of neutrophil granules. Traffic 9, 2151–2164, 10.1111/j.1600-0854.2008.00838.x (2008). 18939952 PMC6363534

[b28] Nilsson M. *et al.* Activation of human polymorphonuclear neutrophils by streptolysin O from *Streptococcus pyogenes* leads to the release of proinflammatory mediators. Thromb Haemost 95, 982–990, 10.1160/TH05-08-0572 (2006). 16732377

[b29] Courtney H. S., Hasty D. L. & Dale J. B. Anti-phagocytic mechanisms of *Streptococcus pyogenes*: binding of fibrinogen to M-related protein. Molecular microbiology 59, 936–947, 10.1111/j.1365-2958.2005.04977.x (2006). 16420362

[b30] Seo H. S. *et al.* Bacteriophage lysin mediates the binding of streptococcus mitis to human platelets through interaction with fibrinogen. PLoS Pathog 6, e1001047, 10.1371/journal.ppat.1001047 (2010). 20714354 PMC2920869

[b31] Thulin P. *et al.* Viable group A streptococci in macrophages during acute soft tissue infection. PLoS Med 3, e53, 10.1371/journal.pmed.0030053 (2006). 16401174 PMC1326258

[b32] Norrby-Teglund A. *et al.* Evidence for superantigen involvement in severe group A streptococcal tissue infections. J Infect Dis 184, 853–860, 10.1086/323443 (2001). 11509997

[b33] Lauth X. *et al.* M1 protein allows Group A streptococcal survival in phagocyte extracellular traps through cathelicidin inhibition. Journal of innate immunity 1, 202–214, 10.1159/000203645 (2009). 20375578 PMC3241932

[b34] Påhlman L. I. *et al.* Soluble M1 protein of *Streptococcus pyogenes* triggers potent T cell activation. Cell Microbiol 10, 404–414, 10.1111/j.1462-5822.2007.01053.x (2008). 17900297

[b35] Mohn H., Le Cabec V., Fischer S. & Maridonneau-Parini I. The src-family protein-tyrosine kinase p59hck is located on the secretory granules in human neutrophils and translocates towards the phagosome during cell activation. Biochem J 309 (Pt 2), 657–665 (1995). 7626033 10.1042/bj3090657 PMC1135781

[b36] Gutkind J. S. & Robbins K. C. Translocation of the FGR protein-tyrosine kinase as a consequence of neutrophil activation. Proc Natl Acad Sci USA 86, 8783–8787 (1989). 2682659 10.1073/pnas.86.22.8783 PMC298374

[b37] Mocsai A. *et al.* Kinase pathways in chemoattractant-induced degranulation of neutrophils: the role of p38 mitogen-activated protein kinase activated by Src family kinases. J Immunol 164, 4321–4331 (2000). 10754332 10.4049/jimmunol.164.8.4321

[b38] Lindmark A., Garwicz D., Rasmussen P. B., Flodgaard H. & Gullberg U. Characterization of the biosynthesis, processing, and sorting of human HBP/CAP37/azurocidin. J Leukoc Biol 66, 634–643 (1999). 10534120 10.1002/jlb.66.4.634

